# Response to “Methodological Considerations in the Pilot Study of a Novel Transcutaneous Peripheral Nerve Stimulation System for Essential Tremor”

**DOI:** 10.5334/tohm.1099

**Published:** 2025-09-24

**Authors:** Richard Dewey, Kelly E. Lyons, Zhen Zhang

**Affiliations:** 1 Parkinson’s Disease and Movement Disorders Center of Boca Raton, Boca Raton, FL, US; 2 University of Kansas Medical Center, Department of Neurology, Kansas City, KS, US; 3 Fasikl Inc., US

We appreciate the author’s comments. This was a pilot study of an AI-controlled, non-invasive therapy for essential tremor (ET). We agree with the author’s comments on the limitations of the study, such as short duration, the lack of blinding, and the subjectivity in the assessments. These limitations have been acknowledged in the Discussion section of the paper. Regarding the inclusion criteria requiring patients to be familiar with smartphones and Wi-Fi, this is a requirement because the device gets real-time adjustment by an AI engine in the cloud. We agree that it could be a limitation for some, especially in countries where smartphones and internet connections are not readily available. However, the Pew Research Center has reported that 91% of adults in the US (the population for this study) own a smartphone, including 79% of people age 65 or above [1]. Therefore, we believe the study results can be generalized to ET patients in the US.

The conclusion of the pilot study was that TPNS showed promise as a safe and effective treatment option for ET patients, which we believe is appropriate.
